# Is Alcohol a Food?—When Should Malt Liquors Be Preferred to Wines and Spirits in the Treatment of Disease? Etc., Etc.

**Published:** 1881-09

**Authors:** H. C. Wood


### IS ALCOHOL A FOOD?—WHEN SHOULD MALT LIQUORS BE PREFERRED TO WINES AND SPIRITS IN THE TREATMENT OF DISEASE? ETC., ETC.

BY H. C. WOOD, M.D.

Before attempting to express briefly an opinion as to the food-value of alcohol, and to marshal the facts upon which such opinion rests, it is proper to define the term "food," lest verbal confusion arise. Some persons may incline to recognize as foods only those substances which either in their intirety or in a more or less altered condition are capable of being formed into the bodily structure. It is plain, however, that this use of the word is too restricted. Of the substances taken daily into the stomach as sustenance only a portion becomes an integrant part of the animal economy, as is shown by the enormous increase in the formation of urea which follows a heavy meal of meat. The appetite for blubber which is produced by exposure to an Artic atmosphere indicates that the system needs an extra supply of fatty food—an indication confirmed by the beneficient effect upon the exposed organism of a highly carbonaceous and liberal diet. The arctic explorer does not fatten, although he may eat enormously of fat. The carbon compound is evidently fuel which is burned up to maintain the bodily heat—i. e., to yield force to maintain the molecular movements of the organism. It is not necessary to elucidate the point further. It is plain that under many circumstances that which is eaten is used not for reconstruction, but for force production.

If the term food be employed in its narrower and, as I think, improper sense, it must be acknowledged that there is no proof that alcohol is a food. The enormous accumulation of fat frequently seen in beer-drinkers, and to a less extent in those who employ dilute alcohol in other form, would seem to indicate that alcohol is capable of being converted into fat, but is probably better explained in other ways.

When the term food is used in its wider and more correct sense, the question "Is alcohol a food?" must receive an answer essentially different from that just given.

From this point of view, any substance which is destroyed in the system, and during the destruction yields force, is a food. The evidence that alcohol fulfils both these conditions is most positive; indeed, so positive, that there is probably no one of those present that would deny at least that alcohol is burnt up in the body; and, if it be burnt up, it must yield heat—i. e. force.

The questions whether alcohol is an economic food, whether it is practically useful as a food, are entirely distinct from that just answered. In regard to its economy, and ounce of good whisky may be estimated to cost about three and one half cents to the man who buys by the gallon. According to the researches of Dupre, it would require about five ounces of lean beef to yield the same amount of force as that produced by the ounce of alcohol: these five ouuces may be fairly estimated as costing at least five cents to the ordinary consumer; so that the advantage is plainly on the side of the spirit. There are, however, various foods cheaper than is lean meat. Tallow at six cents a pound in its burning certainly affords much more of force for the money than does alcohol.

Looking at the matter practically, and not as to a theoretic economy, it is evident that, as alcohol is usually taken and as food is usually eaten, no claim can be made as to the superior cheapness of the beverage. Again, it is notorious that in America almost every one in reasonable health consumes much more food than the system needs, so that any alcohol taken is added to that which is already in excess. In Europe it is different: a large part of the population is underfed, and a modicum of alcohol is a decided food-gain. It must be remembered, also, that as a food alcohol is superior even to lean beef, in that it requires no force to digest it. The coarse food of a European peasant is often worked up by the stomach only by the expenditure of much force; it is also repulsive to the palate. The draught of landwein or the schooner of cheap beer washes down very well the morsel of black bread. Not only by stimulating the stomach does it aid in digestion, but also by readily yielding force to the system it assists in the elaboration of more refractory substances.

Although I hold that the habitual use of alcohol is to well-fed persons not only unnecessary but positively harmful, it seems to me that in many cases of illness and in those periods of life when by reason of age the body waxes weak, alcohol is possessed of great value. Under sixty years of age the daily employment of wine may for most persons be very well discountenanced; but after this period has been reached, I believe the moderate employment of stimulants is very useful. The progressive failure of bodily powers points to the use of a substance which shall aid in digestion and readily supply force. In the later years of life even the narcotic influence of alcohol is of value, easing the restlessness, the slight discomforts, the suffering of nerve-failure incident to failing vitality.

The question whether alcohol has food-value in disease is one not easily answered by positive evidence, because the narcotic properties of the substance are so marked as often to mask its influence as a food, and because we rarely dare to employ alcohol except with an abundance of other food. The principles already outlined are, however, as applicable in disease as in health. Recent researches in fever have determined that the excessive heat-production is dependent upon excessive changes in the stored materials of the body, and it is improbable, though not impossible, that alcohol is capable of taking the place of these, and, by being, as it were, vicariously burnt, saving the tissues. The value of alcohol in low fevers therefore probable depends upon other qualities than its usefulness as a food, although our knowledge of fever-processes is yet so imperfect that it is necessary to speak with great reserve.

In chronic wasting diseases I believe alcohol has an actual food-value, besides being a most powerful aid to the digestion of other food. Arguments upon this point do not seem required, and it is, of course, very difficult to give actual clinical proofs, because we always employ alcohol with other foods and remedies.

The question as to the best method of administering alcohol when it is used for its sustaining powers is of vital interest.

Two general propositions will, I believe, command almost universal assent. First, the alcohol must be given in a dilute form; second, it should be given along with other food. Provided these two rules are observed, I do not think it makes much difference in what form the drug is administered. In chronic diseases malt liquors have both advantages and disadvantages. They represent food and drink, are less apt to be abused than are stronger liquids, and by virtue of their bitterness have some tonic properties; on the other hand they sometimes disagree with the stomach. As they contain some nutritive material, there is perhaps more tendency to administer them apart from food than there should be. The amount of solid constituents in a pint of malt liquor varies from over two and a half ounces of dry residue in the strongest English ales to three quarters of an ounce in the weakest ales and beers. The ales and beers usually drunk in this city probably range from one to two ounces of solid contents to the pint. The nature of much of this solid matter is not known, but albumen, bitter and resinous principles from the hop, earthy salts, grape-sugar, glycerin, and a number of complex acids have been recognized in it. The tendency to grossness seen in beer-drinkers undoubted largely depends upon the solid constituents of the beer which is taken, and seems to me to indicate the proper medical use of malt liquors—namely that they are especially to be employed in wasting diseases, i. e., where there is a tendency to the loss of the bodily fat.

In regard to the choice of malt liquors, I do not think there is any other than what we may call personal grounds for selection. That which suits the palate best usually suits also the stomach best. The choice should always settle upon the ale, porter, or beer which can be used with least inconvenince the stomach; and when all malt liquors produce "biliousness"—i. e., gastro-intestinal derangement—wine or diluted spirits should be substituted. As the malt liquors contain nutritive material, it is less necessary to give food with them than it is with whisky or wines. Nevertheless, it is preferable in most cases that food should be taken with the ale or beer.

